# An automatic semen processing system versus a manual method for semen processing: a randomized, single-blind non-inferiority trial

**DOI:** 10.3389/fendo.2026.1885853

**Published:** 2026-07-22

**Authors:** Qiuxia Yan, Chen Zeng, Guozhi Zhang, Jing Qiao, Huyu Zhou, Zhenjie Zhu, Ling Feng, Xiaoying Zhao, Hong Hu, Piaoyan Zhou, Wanting Huang, Xiuqin Zhou, Lei Li, Cairong Chen, Jianqiao Liu

**Affiliations:** 1 Center for Reproductive Medicine, The Affiliated Qingyuan Hospital (Qingyuan People’s Hospital), Guangzhou Medical University, Guangdong Engineering Technology Research Center of Urinary Continence and Reproductive Medicine, Qingyuan, China; 2 Department of Obstetrics and Gynecology, Center for Reproductive Medicine, Guangdong Provincial Key Laboratory of Major Obstetric Diseases, Guangdong Provincial Clinical Research Center for Obstetrics and Gynecology, Guangdong-Hong Kong-Macao Greater Bay Area Higher Education Joint Laboratory of Maternal-Fetal Medicine, The Third Affiliated Hospital of Guangzhou Medical University, Guangzhou, China; 3 Key Laboratory for Reproductive Medicine of Guangdong Province, The Third Affiliated Hospital of Guangzhou Medical University, Guangzhou, China

**Keywords:** automatic semen processing system, manual method, non-inferiority, progressively motile sperm recovery rate, semen processing

## Abstract

**Background:**

In assisted reproductive technology (ART) laboratories, semen processing is traditionally performed using manual density gradient centrifugation (DGC), which is labor intensive, time consuming, and subject to inter-technician variability. This study aimed to evaluate the effectiveness and repeatability of an automatic semen processing system (Avensen (Shenzhen) Medical Tech Co., Ltd., Shenzhen, China) compared with the standard manual method.

**Methods:**

A total of 110 eligible male participants (aged 18−45 years) were enrolled. Each semen sample was randomized into three 1−mL aliquots, of which two were processed automatically (automated groups 1 and 2) and one manually (manual group). Independent t−tests were used to compare the progressively motile sperm recovery rate between the automated and manual groups. System repeatability was assessed between the two automated groups using the intraclass correlation coefficient, the coefficient of variation, and Bland–Altman analysis.

**Results:**

The automatic system was non-inferior to the manual method in terms of the primary outcome. The progressively motile sperm recovery rate were 42.26% and 42.40% in automated groups 1 and 2, respectively, versus 41.64% in the manual group. The lower 95% confidence limits were -1.39% and -1.37%, both exceeding the -6% non-inferiority margin. Post-processing percentage of progressively motile sperm (PR%) was significantly higher in both automated groups: 62.06% and 62.63% versus 58.83% (P<0.001). No significant differences were found in sperm concentration or total motile count (P>0.05). Repeatability was excellent: the recovery rate had an intraclass correlation coefficient of 0.95 (95% CI: 0.93–0.97) and a mean coefficient of variation of 10.87%. Correlation analysis (r=0.9092), Deming regression (slope=1.0277), and Bland–Altman analysis (mean difference -0.1448%, 95.41% within limits) indicated strong agreement without bias.

**Conclusion:**

The investigated automatic semen processing system is non-inferior to the standard manual method in terms of the progressively motile sperm recovery rate, and the results show high repeatability and consistency. The automatic semen processing system can help technicians reduce their workload, improve work efficiency, and better meet clinical needs.

**Clinical trial registration:**

https://www.chictr.org.cn/showproj.html?proj=274183, identifier ChiCTR2500105131.

## 1 Introduction

Infertility is defined as the inability to conceive after 12 months of regular unprotected intercourse (1). It affects approximately one in six individuals worldwide during their reproductive years, and its prevalence is steadily increasing (2, 3). Etiologies are categorized as female related (e.g., ovulatory disorders, tubal blockage, or endometriosis), male related (obstructive or non-obstructive), or unexplained (4, 5). Assisted reproductive technology (ART) is a key strategy for treating infertility, with an estimated 10–13 million births attributed to ART globally (6). Given the global decline in sperm counts, the demand for ART is expected to increase further (7). Available ART techniques range from intrauterine insemination (IUI) to *in vitro* fertilization (IVF) and intracytoplasmic sperm injection (ICSI) (8). Semen processing is a critical initial step in ART, as sperm quality directly influences clinical pregnancy rates (9). Poor sperm quality has been linked to increased risks of early miscarriage, epigenetic abnormalities, and long-term health issues among offspring (10–12). Sperm damage can arise from intrinsic abnormalities or harmful seminal plasma components, including inflammatory factors, reactive oxygen species, pathogens, and leukocytes, that induce oxidative stress, DNA damage, and cell death (13–16). Thus, the primary goals of semen processing are to select motile, morphologically normal sperm and to remove deleterious components to provide high-quality sperm for assisted reproduction (17).

Semen processing has a standard workflow, including liquefaction, verification, quality assessment, sperm preparation, washing, and resuspension. Density gradient centrifugation (DGC) is the gold standard for sperm preparation (8). It outperforms other methods, such as swim-up, electrophoretic separation, magnetic-activated cell sorting, microfluidic sorting, and hyaluronic acid-binding selection. These methods are less commonly used in routine clinical practice (18–22). DGC separates sperm on the basis of density and motility. Highly motile, denser sperm migrate through the gradient and form a pellet. Meanwhile, immotile or dead sperm and debris remain in the upper layers (23, 24). Despite its widespread use, DGC has several operational limitations. Only one specimen can be processed per workstation at a time to prevent accidental substitution. Manual processing is complex, time consuming, and labor intensive. As the results are highly technician dependent, they are subject to inter-operator variability and limited standardization (6, 25–27). Moreover, each step requires dual-technician verification. Repeated tube opening and pipetting also increase complexity and labor costs. These limitations are becoming increasingly problematic as ART demand and laboratory workloads increase.

Automation has been widely adopted in healthcare. It improves efficiency, reduces human error, ensures consistency, and increases throughput (28–30). However, automation in semen processing has lagged behind. Most current systems are only partially automated. Existing semi-automated devices include MDDS microfluidic chips, IUI-specific automated systems, and the BLASTO-chip. These devices offer stepwise improvements but still require manual intervention for washing, verification, or other critical procedures (17, 31, 32). These incremental enhancements do not address the fundamental bottleneck in clinical ART laboratories. Workflows remain largely manual, making standardization and scale-up difficult as demand increases. The automatic discontinuous density gradient centrifugation processing system for semen, namely Avensen (Shenzhen) Medical Tech Co., Ltd., Shenzhen, China, represents a significant advance. It is the first instrument capable of completing the entire ART semen processing workflow automatically.​The system uses a discontinuous DGC method to optimize and select sperm. It performs every step automatically, from preparing solutions and verifying samples to loading, centrifugation, washing, and resuspension. Furthermore, it can process 12 semen samples simultaneously. Integrated radio-frequency identification (RFID) and optical character recognition (OCR) verification systems prevent sample misidentification throughout the process. However, owing to a lack of clinical trial validation, concerns remain regarding the efficacy and stability of the system.

Therefore, the primary objective of this study was to evaluate the efficacy and consistency of this automated semen processing system. To this end, we conducted a single-center, randomized, single-blind, self-paired non-inferiority trial to compare the sperm processing outcomes of this system to those of the standard manual DGC method. The secondary objective was to assess the system’s repeatability.

## 2 Methods

### 2.1 Study design

This single-center, randomized, single-blind, self-paired non-inferiority trial compared the sperm processing outcomes of an automated system against those of the standard manual density gradient centrifugation (DGC) method. To assess repeatability, the same instrument processed the same sample twice, designated automated group 1 and automated group 2.

### 2.2 Participants

Male participants aged 18–45 years (inclusive) were enrolled. The inclusion criteria included liquefaction within 60 min and normal viscosity (thread ≤2 cm), a semen volume ≥ 3.2 mL, percentage of progressively motile sperm (PR%) ≥ 32%, a sperm concentration ≥ 15 × 10^6^/mL, an abstinence interval of 2–7 days before collection, and signed informed consent. These thresholds were based on the WHO reference values (8). The exclusion criteria included inability to provide an adequate sample, inability to understand or comply with study procedures, and any other condition considered exclusionary by the investigator.

### 2.3 Randomization

The screening identifiers followed the format “S” plus three Arabic digits (e.g., S001). Each raw semen sample received a unique traceable code to ensure linkage to its donor. A statistician generated a block randomization sequence using SAS software (version 9.4) with a 1:1:1 allocation ratio across three groups: manual group, automated group 1, and automated group 2. On the basis of this sequence and the order of enrolment, a laboratory technician obtained the corresponding random identifier and group assignment for each sample. Following the initial analysis, the well-mixed semen sample was divided into three separate 1-mL aliquots, which were allocated to their respective processing groups according to the randomization sequence.

### 2.4 Procedures

#### 2.4.1 Screening and sample allocation

**Figure 1:**
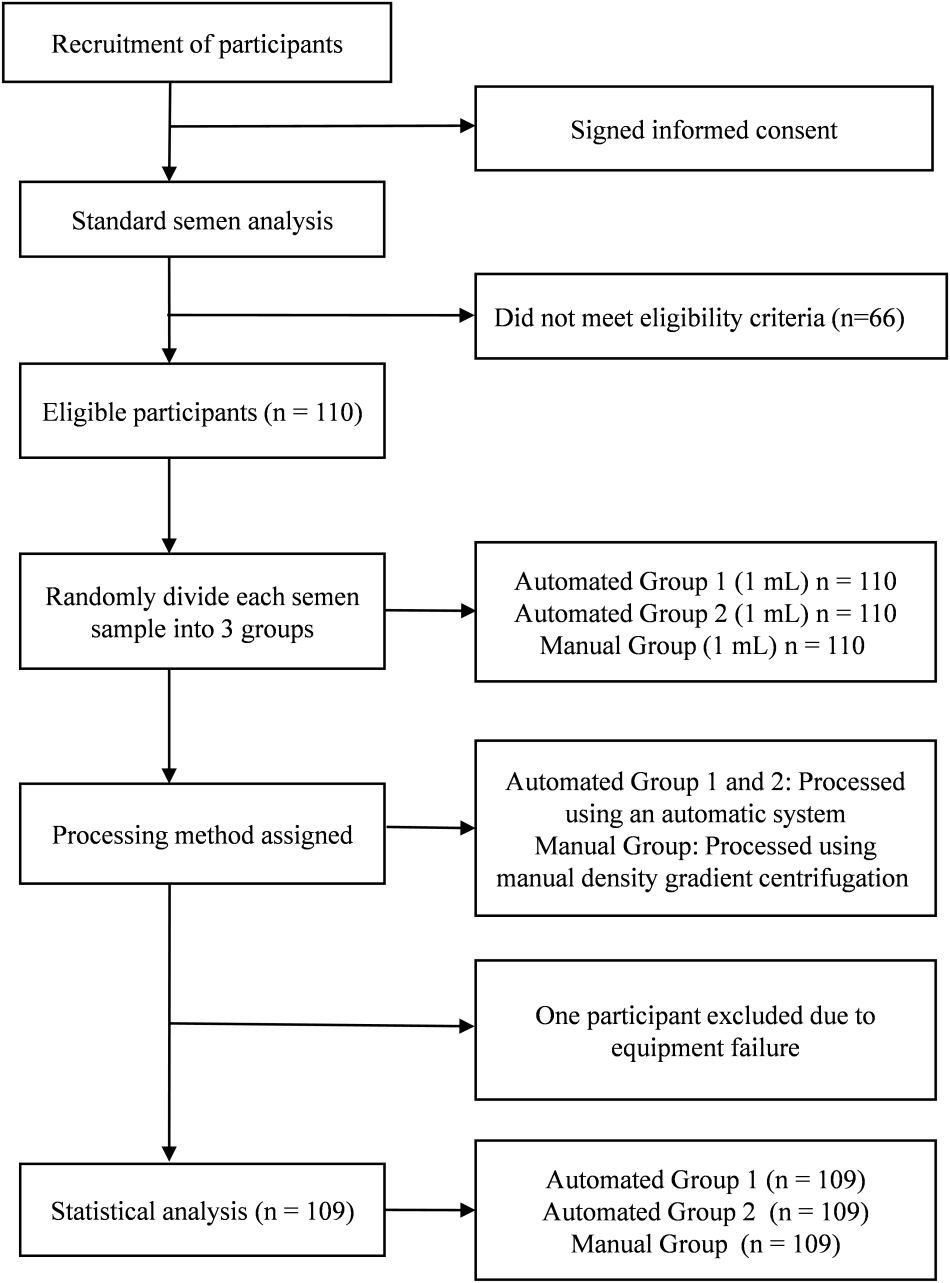
Study flow diagram.

All participants provided written informed consent prior to any screening activities. Eligibility was confirmed against predefined inclusion and exclusion criteria, and a standard semen analysis was performed, including assessment of liquefaction, semen volume, sperm concentration, and PR%. Each raw semen sample received a unique traceable code to ensure linkage to its donor. The samples were then allocated according to the randomization procedure described in section 2.3. **Figure 1** outlines the workflow.

#### 2.4.2 Manual DGC processing

For manual processing, all solutions were prepared in advance and equilibrated for 20 minutes at room temperature. Semen was manually applied to a slide for microscopic examination. A 15-mL centrifuge tube (Cat No. 352099, Falcon, Mexico) was loaded with 1 mL of 90% and 1 mL of 45% density gradient media (Vitrolife, Sweden) to form a distinct interface. One milliliter of liquefied semen was then carefully layered over the gradient. Centrifugation was performed at 300 × g for 20 minutes (Centrifuge 5804; Eppendorf SE, Germany). The supernatant was discarded, leaving approximately 0.5 mL of sediment, which was gently resuspended in 2 mL of G-IVF Plus medium (Vitrolife, Sweden). A second centrifugation was carried out at 200 × g for 5 minutes.

#### 2.4.3 Automated system processing

For automated processing, the automatic semen processing system (Avensen (Shenzhen) Medical Tech Co., Ltd., Shenzhen, China) performed all steps without manual intervention. Reagents, empty centrifuge tubes, and sample tubes with RFID tags were placed in designated positions by the operator. The system automatically aspirated the semen, dispensed it onto a slide, and then prompted the operator to retrieve the slide for microscopic examination. The RFID information was automatically read, and a label was printed and applied to a new centrifuge tube. The robotic arm automatically dispensed liquids in a programmed sequence, tilting the tube during addition to ensure a clear interface between the gradient layers. The tube was placed into the centrifuge, automatically balanced, and centrifuged at 300 × g for 20 minutes. After centrifugation, the tube was removed by the robotic arm and photographed for documentation. The upper supernatant was automatically aspirated, and the sperm pellet at the bottom was retained. Resuspension and washing were subsequently performed by the robotic arm. The tube containing the final product was placed on the product carrier, and the system signaled completion. The entire process was conducted under cleanroom conditions with temperature control. Abnormal samples were automatically diverted, and all procedures were logged by the system to ensure traceability.

#### 2.4.4 Blinding and analysis

To maintain blinding, the processed samples were re-coded by a technician who was not involved in the subsequent analysis. A master list linking the original identifiers to the blinding codes was securely maintained. The blinded samples were then transferred to analysts who were unaware of sample origin or group assignment. Unblinding was conducted by a sample custodian only after all analyses were complete.

#### 2.4.5 Sample analysis

Semen volume was measured by gravimetric analysis. After complete liquefaction at 37°C for 30–60 minutes. For both manual and automated groups, sperm concentration and PR% were analyzed by a computer-assisted sperm analysis (CASA) system (version SCA-P-H-02; Microptic Co., Spain) (33). For CASA analysis, a 5-μL aliquot of the liquefied sample was placed on a pre-warmed disposable counting chamber (20 μm depth) (Cat#Al-20-4; AIYX Medical Technology Co., Ltd., China), and examined under ×100 magnification. At least 200 spermatozoa were evaluated per replicate. Each sample was tested twice, and the average of the two measurements was used for analysis. All analytical procedures followed the World Health Organization (WHO) laboratory manual for the examination and processing of human semen (5th edition) (8).

### 2.5 Outcome measures

The primary outcome was the progressively motile sperm (PR) recovery rate, a standard metric for evaluating the efficacy of semen processing optimization. A higher recovery rate of PR indicates a greater yield of functional sperm. It was calculated using the following formula: recovery rate of PR (%) = (total count post-processing PR/total count pre-processing PR) × 100%. The total counts, both pre- and post-processing, were calculated as concentration × PR% × volume.

The secondary outcome measures included post-processing sperm concentration and PR%, both of which were directly measured using a CASA system. Additionally, the total count of progressively motile sperm after processing was calculated as: concentration × PR% × volume. The selection of all outcome measures followed the standardized methodologies and recommendations of the WHO manual (5th edition) (8).

### 2.6 Sample size calculation

According to preliminary data (mean progressively motile sperm recovery rate of 0.3 for manual processing) and a non-inferiority design (non-inferiority margin of 0.06, α = 0.025, β = 0.2), the sample size was calculated as 110 participants after accounting for a 10% dropout rate. Detailed calculations are provided in the Supporting Information.

### 2.7 Statistical analysis

All the data were analyzed using statistical software (SAS, version 9.4). For quantitative variables, normality was assessed using the Shapiro-Wilk test. Variables with a normal distribution are expressed as the mean ± SD, and a paired t−test was used for comparisons between groups. Quantitative data with a non−normal distribution are summarized as the median and interquartile range [*M (Q1, Q3)*], with group comparisons based on the Wilcoxon signed−rank test. Categorical variables are summarized as frequencies and percentages, and the paired chi−square test was used to assess intergroup differences. The intraclass correlation coefficient (ICC) was calculated to evaluate the consistency of measurements from the same subject using the automatic system. In addition, the mean coefficient of variation (CV) was determined to assess within-subject variability. To assess the repeatability of the automated system, Pearson correlation and Deming regression were performed on the data from the two automated processing runs, and Bland–Altman analysis was used to evaluate their agreement. We performed two-tailed statistical tests for all comparisons. *P* < 0.05 was considered to indicate statistical significance.

## 3 Results

### 3.1 Participant baseline characteristics

In summary, of the 110 enrolled participants, 109 completed the trial. One participant was excluded because of equipment failure (pipette origin position not updated). The demographic and semen characteristics of the study population are summarized in **Table 1**. The mean age was 31.91 ± 4.75 years, and all participants had normal semen liquefaction and viscosity. These baseline values indicate that the study population was typical for men undergoing semen analysis, supporting the generalizability of the findings.

**Table 1:** Baseline characteristics of the participants.

Analysis variable	Results
Category/Statistic	N, Total=109
Gender, N(%)	
N	109
Male	109(100.0)
Age (years)	
N	109
Mean ± SD	31.91 ± 4.75
Median(Q1, Q3)	32.00(28.00,35.00)
Min∼Max	21.00∼42.00
Semen liquefaction status, N(%)	
N	109
Normal	109(100.0)
Abnormal	0(0.0)
Semen viscosity, N(%)	
N	109
Normal	109(100.0)
Abnormal	0(0.0)
Semen volume (mL)	
N	109
Mean ± SD	4.45 ± 1.16
Median(Q1, Q3)	4.20(3.50,4.90)
Min∼Max	3.20∼9.90
Concentration (×10^6^/mL)	
N	109
Mean ± SD	66.95 ± 43.57
Median(Q1, Q3)	53.00(35.80,99.95)
Min∼Max	15.40∼261.10
PR%	
N	109
Mean ± SD	45.90 ± 9.55
Median(Q1, Q3)	45.20(37.75,52.90)
Min∼Max	32.00∼80.50
Abstinence time prior to sampling (days)	
N	109
Mean ± SD	4.39 ± 1.51
Median(Q1, Q3)	4.00(3.00,5.00)
Min∼Max	2.00∼7.00

### 3.2 Comparison of the automated system and the manual method

The mean post-processing progressive motile sperm recovery rates were 42.26% in automated group 1 versus 41.64% in the manual group (P > 0.05), and 42.40% in automated group 2 versus 41.64% in the manual group (P > 0.05). Overall, the automated system was non-inferior to the manual DGC method in terms of the primary outcome, progressively motile sperm recovery rate (**Table 2**). The lower confidence limits for both automated groups were well above the non-inferiority margin of -6%, confirming that the automated system performs at least as well as the manual method. Regarding secondary outcomes, the automated system significantly improved post-processing PR% (**Table 3**, P < 0.001). In contrast, sperm concentration and total progressively motile sperm count did not differ significantly differ between groups (P > 0.05). Taken together, these results suggest that automation enhances sperm motility without compromising other quality parameters.

**Table 2:** Non-inferiority analysis of progressively motile sperm recovery rate after semen processing.

Comparison group	Prespecified margin	Statistical method	Mean difference (%)	95% CI lower limit (%)	95% CI upper limit (%)	Conclusion
Automated group 1 - manual group	-6%	Paired t-test	0.62	-1.39	2.63	The test device is non-inferior to the manual method.
Automated group 2 - manual group	-6%	Paired t-test	0.76	-1.37	2.89	The test device is non-inferior to the manual method.

**Table 3:** Comparison of sperm parameters between automated and manual groups.

Analysis variable	Automated group 1 N=109	Automated group 2 N=109	Manual group N=109	P value (Automated group 1 - manual group)	P value (Automated group 2 - manual group)
Concentration (×10^6^/mL)				0.314	0.253
N	109	109	109		
Mean ± SD	35.53 ± 41.57	35.48 ± 41.71	36.33 ± 44.60		
Median(Q1, Q3)	27.30(17.85,40.10)	27.90(18.10,41.18)	27.65(18.83,42.85)		
Min∼Max	5.65∼343.30	4.75∼354.55	4.95∼386.85		
PR%				<0.0001	<0.0001
N	109	109	109		
Mean ± SD	62.06 ± 14.52	62.63 ± 14.59	58.83 ± 15.83		
Median(Q1, Q3)	61.20(55.63,72.15)	64.45(53.30,72.10)	58.85(49.28,69.98)		
Min∼Max	25.70∼96.45	20.55∼97.15	15.60∼97.05		
Total progressive motile sperm count (×10^6^)				0.705	0.33
N	109	109	109		
Mean ± SD	13.02 ± 20.18	12.94 ± 19.11	12.72 ± 20.88		
Median(Q1, Q3)	8.36(5.06,13.51)	8.76(5.02,14.92)	8.55(4.72,13.41)		
Min∼Max	1.27∼149.96	1.09∼150.14	0.87∼165.58		

### 3.3 Repeatability and consistency of the automatic system

The automated system showed strong repeatability and consistency across two independent processing runs of the same samples. The following analyses support this conclusion.

#### 3.3.1 Intraclass correlation coefficient analysis

To assess the repeatability and consistency of the automatic system, a one−way random−effects model based on absolute agreement and average measures was applied. The ICC calculated for PR% between automated group 1 and automated group 2 was 0.95 (95% CI: 0.93 to 0.97). The test device was confirmed to have strong consistency, as the lower limit of the 95% CI (0.93) exceeded the pre-defined threshold of 0.80 (**Supplementary Table 1**).

#### 3.3.2 Coefficient of variation for repeated measurements

The CV fell within the acceptable range (mean CV = 10.87%; **Supplementary Table 2**). This value was below the WHO threshold of 15% (8). Therefore, the automated system meets the standard for repeatability.

#### 3.3.3 Correlation analysis

The PR% values from automated group 1 and automated group 2 were log transformed, after which the bivariate normality assumption was satisfied. Pearson correlation analysis was then performed. A strong correlation coefficient (r) of 0.9092 was obtained (95% CI: 0.8689 to 0.9365). The coefficient of determination (r²) was 0.8267. These results indicated a strong correlation between the two sets of measurements from the automated device, confirming the consistency of its output (**Supplementary Table 3**).

#### 3.3.4 Regression analysis

**Figure 2:**
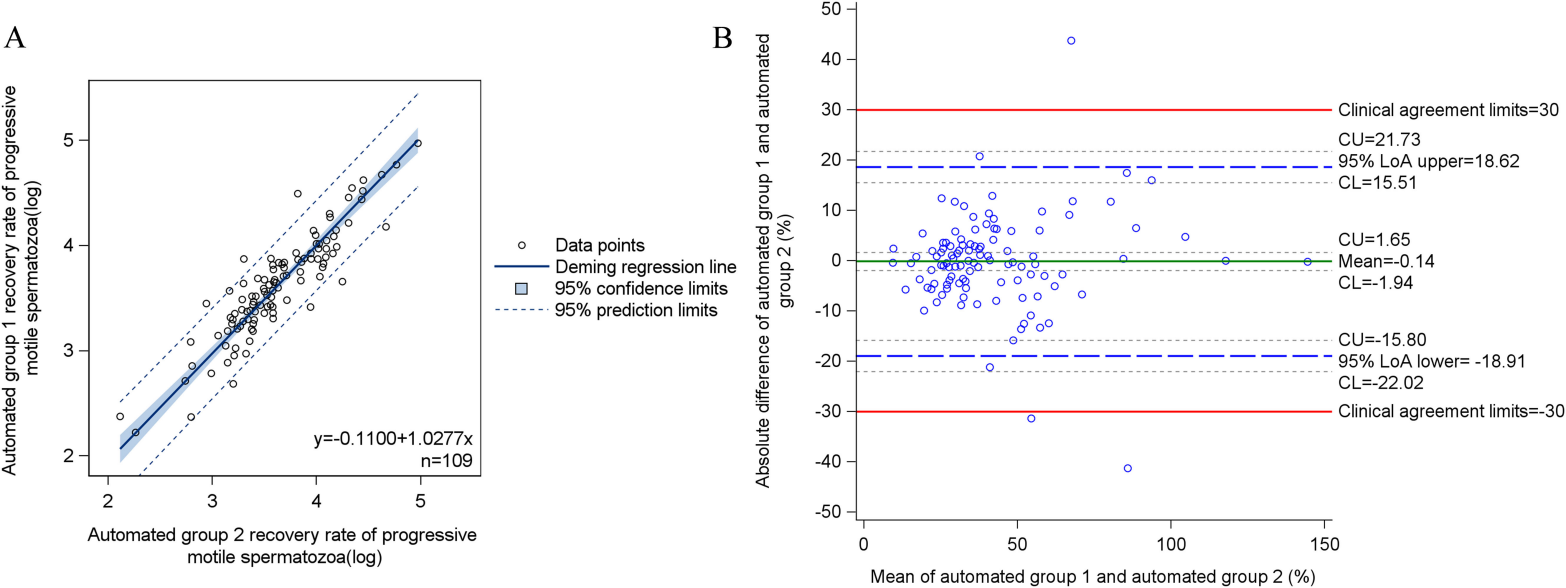
Reproducibility between automated semen analysis platforms. **(A)** Linear regression plot for automated group 1 versus automated group 2. **(B)** Bland–Altman plot of absolute differences between automated group 1 and automated group 2. The solid green line represents the mean bias (-0.14%; 95% CI: -1.94 to 1.65). The dashed blue lines represent the 95% limits of agreement (LoA) (lower: -18.91% [95% CI: -22.02 to -15.80]; upper: 18.62% [95% CI: 15.51 to 21.73]). Among the 109 data points, 104 (95.41%) fell within the LoA. The solid red lines indicate the pre-defined clinical agreement limits (± 30%).

Deming regression was performed on the log-transformed PR% data from automated group 1 and automated group 2. Deming regression confirmed the absence of systematic bias between the two runs (**Figure 2A**; **Supplementary Table 4**). The 95% CI for the slope included 1, and the CI for the intercept included 0, providing no evidence of proportional or fixed bias.

#### 3.3.5 Bland–Altman analysis

The differences in PR% between automated group 1 and automated group 2 were approximately normally distributed (**Supplementary Figure 1**). Bland–Altman analysis indicated no systematic bias and confirmed good repeatability (**Figure 2B**; **Supplementary Table 5**). The mean difference was -0.14% (95% CI: -1.94% to 1.65%), and 95.41% of the data points fell within the 95% limits of agreement(LoA). This meets typical agreement standards and confirms that the automated system yields reproducible results across repeated runs.

## 4 Discussion

In ART, the main goal of semen processing is to maximize the recovery of high−quality sperm. This is achieved by removing immotile sperm, abnormal cells, leukocytes, and seminal plasma components that may hinder fertilization (33, 34). This comprehensive processing ultimately aims to increase the clinical pregnancy rate. An ideal automated system should efficiently isolate motile sperm without causing damage and should be rapid and easy to operate. Currently, no single device meets all these criteria.

Conventional techniques such as DGC and the swim−up method are predominantly manual (8). These methods, characterized by operator dependence, time consumption, and labor intensity, also exhibit limited repeatability. In contrast, the automatic system provides integrated functions, including automated sample preparation, dual verification protocols, customizable centrifugation settings, automated documentation, and electronic medical record connectivity. This standardizes workflow, minimizes human error, enables quality control, and reduces labor requirements.

This study provides the first systematic evaluation of an automated sperm processing system against standard manual methods in a single-center, randomized, single-blind non-inferiority trial. The results indicated the non-inferiority of the automatic system in terms of the key efficacy metric, namely, progressively motile sperm recovery rate (see **Table 2**). These findings establish that automated processing can replace manual operation without compromising sperm recovery efficiency. Inter-operator variability in recovery rates has been widely reported (25–27). This variability was closely associated with factors such as sample processing time, centrifugal force, number of washing steps, and pipetting accuracy. In contrast, the present study demonstrated high concordance between the two automated groups (ICC = 0.95), with the mean CV for recovery rate of PR well below the prespecified 15% threshold. Furthermore, Deming regression and Bland–Altman analyses confirmed the absence of significant systematic bias between the two automated groups. Taken together, these findings confirm that the automated device can serve as a reliable alternative to manual processing, helping to reduce the inter-laboratory variability traditionally attributable to operator-dependent technical differences. The superior repeatability of the automatic system is primarily attributable to its standardized and automated design, which eliminates the variations in technique, applied force, and environmental interference that are inherent to manual processing.

To assess whether the automated system induces sperm damage, we evaluated the post-processing semen concentration, PR%, and total progressively motile sperm count. Notably, the PR% values in both automated groups were significantly greater than that in the manual group (P < 0.001; **Table 3**), with an increase of more than 3%. Compared with the manual group, both automated groups had a slightly higher mean total progressively motile sperm count and a slightly lower semen concentration. However, neither difference reached statistical significance. These findings suggested that this automatic semen processing system might slightly reduce total cell density while decreasing seminal plasma impurities and abnormal sperm. However, it demonstrated advantages over conventional manual processing in maintaining sperm motility or selecting sperm quality. This advantage was considered non-random and likely arises from three factors. First, the system eliminates inconsistencies inherent to the manual technique. Manual processing relies on handheld instruments, where variations in instantaneous force and attention introduce uncontrollable interference, leading to pronounced interoperator variability (27, 35). In marked contrast, the CV for the recovery rate of PR in the automated groups (10.9%) was substantially lower than the reported range for manual operations (17%–127%) (35). This difference demonstrates the fundamental advantage of automated workflows in terms of operational stability. Second, temperature fluctuations and air exposure are difficult to avoid in traditional open-bench operations and directly impair sperm motility (36–39). These risks are eliminated by the fully enclosed, thermostatically controlled (37 °C) environment in the automatic system. Finally, this system shortens processing time through programmed procedures, limits contact between sperm and sources of reactive oxygen species present in seminal plasma (such as leukocytes and abnormal sperm), and thereby decreases sperm motility loss caused by oxidative stress (40).

The baseline characteristics of our study population closely resemble those reported in a large-scale clinical trial on DGC published within the past five years (41). This agreement that our cohort was representative and supports the generalizability of our findings to similar clinical settings. Furthermore, the prespecified dropout rate for this trial was 10%. The actual attrition was only one participant (approximately 0.9%), substantially lower than anticipated, reflecting the good quality control of our study. Clinically, we found that the automated system can reliably replace the manual technique for routine semen processing, offering improved consistency, increased throughput, and reduced sample misidentification risk. Future studies should validate its performance in multicenter settings, evaluate its impact on clinical endpoints such as pregnancy and live birth rates, and test its utility with abnormal semen samples.

These strengths notwithstanding, several limitations exist in the present study. First, although the single−center design supports operational standardization, it may restrict the applicability of the findings to other laboratory environments. Second, the inclusion criteria excluded populations such as those with oligozoospermia; therefore, the performance of the device with abnormal semen samples requires further validation. Third, the primary endpoint was limited to the progressively motile sperm recovery rate. Further evaluation is needed to assess the effects on key clinical endpoints, such as the fertilization rate, clinical pregnancy rate, and live birth rate.

## 5 Conclusions

In conclusion, the automated semen processing system is non-inferior to the standard manual method in terms of efficacy and produces highly reproducible results. The implementation of this system facilitates the standardization and automation of semen processing. It holds significant potential for enhancing the operational efficiency and service quality of assisted reproductive technology laboratories thereby reducing the need for trained laboratory personnel to process the semen manually.

## Data Availability

The raw data supporting the conclusions of this article will be made available by the authors, without undue reservation.
