# Supplementary material for: An automatic semen processing system versus a manual method for semen processing: a randomized, single-blind non-inferiority trial
Source: Front Endocrinol (Lausanne). 2026 Jul 22;17:1885853. doi: 10.3389/fendo.2026.1885853 (PMC13437289; doi:10.3389/fendo.2026.1885853)
Supplement: Supplementary file 1 [file SupplementaryFile1.docx]

**Supplementary Materials**

Sample size calculation

Table S1. Intraclass correlation coefficient analysis of the progressively motile sperm recovery rates (%)

Table S2. Coefficient of variation for repeated measures of progressively motile sperm recovery rates

Table S3. Results of the correlation analysis between automated group 1 and automated group 2

Table S4. Parameters of the regression equation

Table S5. Bland‒Altman analysis results for automated group 1 versus automated group 2

Figure S1. Histogram of the differences between automated group 1 and automated group 2 (raw data)

**Sample size calculation**

The sample size for the non-inferiority comparison between the automatic system and the manual method was calculated based on our preliminary data. The mean progressive motile sperm recovery rate for the manual method was 0.30 in our reproductive center, and we hypothesized an equal mean (0.30) for the test device. The non-inferiority margin was set at 0.06 (control minus test device). Given an estimated pooled standard deviation of 0.15, a one-sided alpha of 0.025, and a beta of 0.20 (80% power), a minimum of 99 evaluable participants was required. Considering an estimated 10% dropout rate, a total of 110 participants were needed. The sample size for assessing the repeatability of the automated device was determined separately. Two measurements were obtained from the same sample using the device. An intraclass correlation coefficient (ICC) of 0.80 was set as the target lower limit for an acceptable level of agreement. The expected ICC was estimated to be 0.90. With a two-sided alpha of 0.05 and a beta of 0.10 (90% power), a minimum of 63 participant samples was required for the repeated measurements. To accommodate a potential 10% sample loss, the target was increased to at least 70 participants.

The larger of the two calculated sample sizes, 110 participants, was selected as the final sample size for the trial to ensure both primary objectives were adequately powered.

**Table S1. Intraclass correlation coefficient analysis of the progressively motile sperm recovery rates (%)**

| **Model** | **Type** | **Comparison group** | **Preset standard** | **ICC point estimate** | **95% CI lower limit** | **95% CI upper limit** | **Conclusion** |
| --- | --- | --- | --- | --- | --- | --- | --- |
| One-way random | Average measures | Automated group1 - automated group 2 | 0.8 | 0.95 | 0.93 | 0.97 | The test device demonstrates strong repeatability. |

**Table S2. Coefficient of variation for repeated measures of progressively motile sperm recovery rates**

| **Comparison group** | **Difference between the two groups** | | **Mean coefficient of variation (%)** |
| --- | --- | --- | --- |
|  | **Mean difference (%)** | **Standard deviation of the difference (%)** |  |
| Automated group 1 - automated group 2 | -0.145 | 9.57 | 10.87 |

**Table S3. Results of the correlation analysis between automated group 1 and automated group 2**

| **N** | **Correlation coefficient (r)** | **Coefficient of determination (r²)** | **95% confidence interval** | **P value** | **Method** |
| --- | --- | --- | --- | --- | --- |
| 109 | 0.9092 | 0.8267 | 0.8689～0.9365 | <0.0001 | Pearson correlation analysis |

**Table S4. Parameters of the regression equation**

| **Variable** | **Parameter estimate** | **Standard error** | **t value** | **P value** | **95% confidence interval** |
| --- | --- | --- | --- | --- | --- |
| Intercept (a) | -0.1100 | 0.1767 | -0.6226 | 0.5335 | -0.4603～0.2403 |
| Slope (b) | 1.0277 | 0.0480 | 0.5773 | 0.5638 | 0.9326～1.1228 |

**Table S5. Bland‒Altman analysis results for automated group 1 versus automated group 2**

| **Analysis** | **Item** | **Point estimate** | **95% CI lower limit** | **95% CI upper limit** | **Number (%) below lower LoA)** | **Number (%) above upper LoA** | **Number (%) within LoA** |
| --- | --- | --- | --- | --- | --- | --- | --- |
| absolute bias | Mean difference | -0.1448 | -1.9420 | 1.6524 | 3(2.75) | 2(1.83) | 104(95.41) |
|  | Lower limit of agreement | -18.9080 | -22.0208 | -15.7952 | / | / | / |
|  | Upper limit of agreement | 18.6185 | 15.5056 | 21.7313 | / | / | / |

LoA denotes the limits of agreement, calculated as the mean difference ± Z(1-α/2) × standard deviation of the differences. The 95% confidence intervals for the upper and lower LoA are calculated as LoA ± Z(1-α/2) × SQRT(3 × (standard deviation)²/n).

**Figure S1. Histogram of the differences between automated group 1 and automated group 2 (raw data)**
